# From Delegation to Measurable Safety: A Narrative Review and Core Outcome Framework for Evaluating Physician Assistant Integration in German Ambulatory Care

**DOI:** 10.7759/cureus.113832

**Published:** 2026-08-02

**Authors:** Stefan Bigge

**Affiliations:** 1 Dermatology, Praxis Bigge Ungerechts, Cologne, DEU

**Keywords:** ambulatory care, clinical supervision, diagnostic safety, germany, implementation science, outcome measurement, patient safety, physician assistants

## Abstract

Physician assistants (PAs) are increasingly considered for physician-led ambulatory care teams in Germany, but activity, satisfaction, or direct labor cost alone cannot establish safe integration. This structured narrative review searched PubMed/MEDLINE in seven focused PA-specific blocks and supplemented the search with two documented Google Scholar sensitivity searches. After deduplication, all 1,508 unique records were screened for evidence concerning safety, diagnostic processes, supervision, role transparency, implementation, workload, and German transferability. Sources used in the synthesis were appraised for design, setting, case mix, supervision ascertainment, outcome validity, and directness. The evidence supports feasibility and positive patient or physician experience in selected, supervised, and often protocolized pathways, but it remains heterogeneous and does not establish equivalence across undifferentiated ambulatory case mix. Case complexity, physician supervision time, escalation performance, role understanding, and downstream utilization are frequently unmeasured. An evidence-informed framework is therefore proposed with nine domains: patient and diagnostic safety, diagnostic process quality, escalation and red-flag handling, supervision burden and hidden physician work, role transparency and patient understanding, patient experience, workflow and productivity, economic and resource impact, and implementation, governance, team effects, and sustainability. Tier 1 contains mandatory safety and implementation indicators, whereas Tier 2 contains extended service and economic outcomes. Every report should stratify results by setting, diagnostic stage, case complexity, supervision model, and PA experience. The framework is an author-proposed starting structure, not a validated core outcome set. German multi-stakeholder consensus, prospective feasibility testing, and validation are required before it can support benchmarking or policy thresholds.

## Introduction and background

A recent review proposed a safety-oriented supervision and delegation framework for integrating physician assistants (PAs) into German ambulatory care [1]. The remaining methodological problem is how a practice, researcher, or regulator can determine whether a specific implementation is safe, effective, transparent, and sustainable. Appointment volume may rise while physician interruptions, diagnostic corrections, or downstream use also rise. Without predefined denominators and contextual variables, each observation can be reported as success while the net clinical effect remains uncertain.

This gap is particularly important in Germany, where PA work remains physician-led delegation rather than independent substitution. Official guidance keeps final diagnostic and therapeutic responsibility with the physician and links delegated activity to the individual PA's competence, the clinical context, and supervision [2,3]. German publications describe heterogeneous activities and potential opportunities in outpatient care, but also unresolved legal, organizational, refinancing, and role-boundary questions [4-7]. Direct German evidence on ambulatory safety, diagnostic outcomes, and net physician workload remains limited.

International evidence is encouraging but conditional. Recent UK reviews found few eligible primary-care studies, small numbers of PAs, and no direct study of safety incidents [8,9]. A broader comparative review found the most favorable evidence under direct supervision or in post-diagnostic care, but did not support extrapolation to indirectly supervised, undifferentiated pre-diagnostic care [10]. These findings make PA care a context-dependent service intervention rather than a uniform exposure.

The objective of this review is to translate that evidence gap into a measurable core outcome framework and minimum dataset for German ambulatory care. Specifically, it identifies why conventional PA outcomes are insufficient when reported alone, compares supportive and contradictory findings, defines a two-tier set of outcome domains, standardizes supervision terminology, and proposes a phase-specific pilot model. The novelty is the linkage of implementation fidelity to diagnostic safety, role transparency, hidden physician work, and downstream utilization. The framework is explicitly an author-proposed, evidence-informed structure; it has not undergone stakeholder consensus and is not a validated core outcome set.

## Review

Literature selection and analytical approach

The final literature search was completed on July 27, 2026. PubMed/MEDLINE was searched in seven focused PA-specific blocks covering ambulatory outcomes and case mix, supervision and physician work, onboarding, delegation, governance, and scope, patient understanding and role transparency, diagnostic safety and escalation, and Germany. Broad methodological searches used in the submitted version were replaced by these narrower blocks so that every retrieved PubMed record could be screened. The common PA concept combined the Medical Subject Heading "Physician Assistants" with title/abstract variants of physician assistant and physician associate. Table 1 summarizes each search block and its retrieval count. In total, 52 sources informed the narrative synthesis [1-52]. The full reproducible syntax is provided in the Appendices.

**Table 1:** Final focused search strategy and analytical contribution Searches were completed on July 27, 2026. PA set: ("Physician Assistants"[Mesh] OR "physician assistant"[tiab] OR "physician assistants"[tiab] OR "physician associate"[tiab] OR "physician associates"[tiab]). All 1,533 PubMed records were exported and screened; 1,308 were unique after within-source deduplication. Google Scholar counts are captured displayed positions, not approximate total-result estimates. Source: Original table created by the author.

Block	Search concept and logic	Retrieved	Contribution
P1	PA set AND primary/ambulatory/general practice terms AND targeted outcome, safety, quality, case-mix, reconsultation, referral, workload, or cost terms	132	Direct ambulatory outcomes and case mix [20-23]
P2	PA set AND (supervis* OR clinical/physician oversight OR supervision burden OR supervising physician OR physician time OR workload)	330	Supervision exposure and hidden physician work [24-27]
P3	PA set AND (onboarding OR orientation OR preceptorship OR mentorship OR transition to practice OR role clarification)	336	Onboarding, competence progression, and implementation [26,28-30]
P4	PA set AND (delegat* OR governance OR scope of practice/responsibility OR professional role OR role clarity OR task shifting)	292	Delegation, governance, and scope heterogeneity [24,31-34]
P5	PA set AND (patient/public understanding or perception OR role confusion/identification OR transparency OR satisfaction)	208	Role transparency and patient experience [35-39]
P6	PA set AND (red flag* OR escalat* OR failure to escalate OR diagnostic error/delay OR delayed/missed diagnosis)	40	Diagnostic safety and escalation [14,15,40-42]
P7	PA set AND ("Germany"[Mesh] OR Germany/German/Germans/Deutschland[tiab])	195	German transferability and governance [2-7]
GS1	intitle:"physician assistant" "primary care" safety	200 positions	Sensitivity search and source chaining
GS2	intitle:"physician associate" "primary care" safety	53 positions	Sensitivity search and source chaining

Two supplementary Google Scholar sensitivity searches were documented exactly as intitle:"physician assistant" "primary care" safety and intitle:"physician associate" "primary care" safety. The first 200 displayed positions were captured for the assistant query in a single session, and all 53 displayed positions were captured for the associate query. These searches were used only to identify additional or grey literature candidates; Scholar's approximate total and relevance ordering were not treated as reproducible denominators.

**Figure 1:**
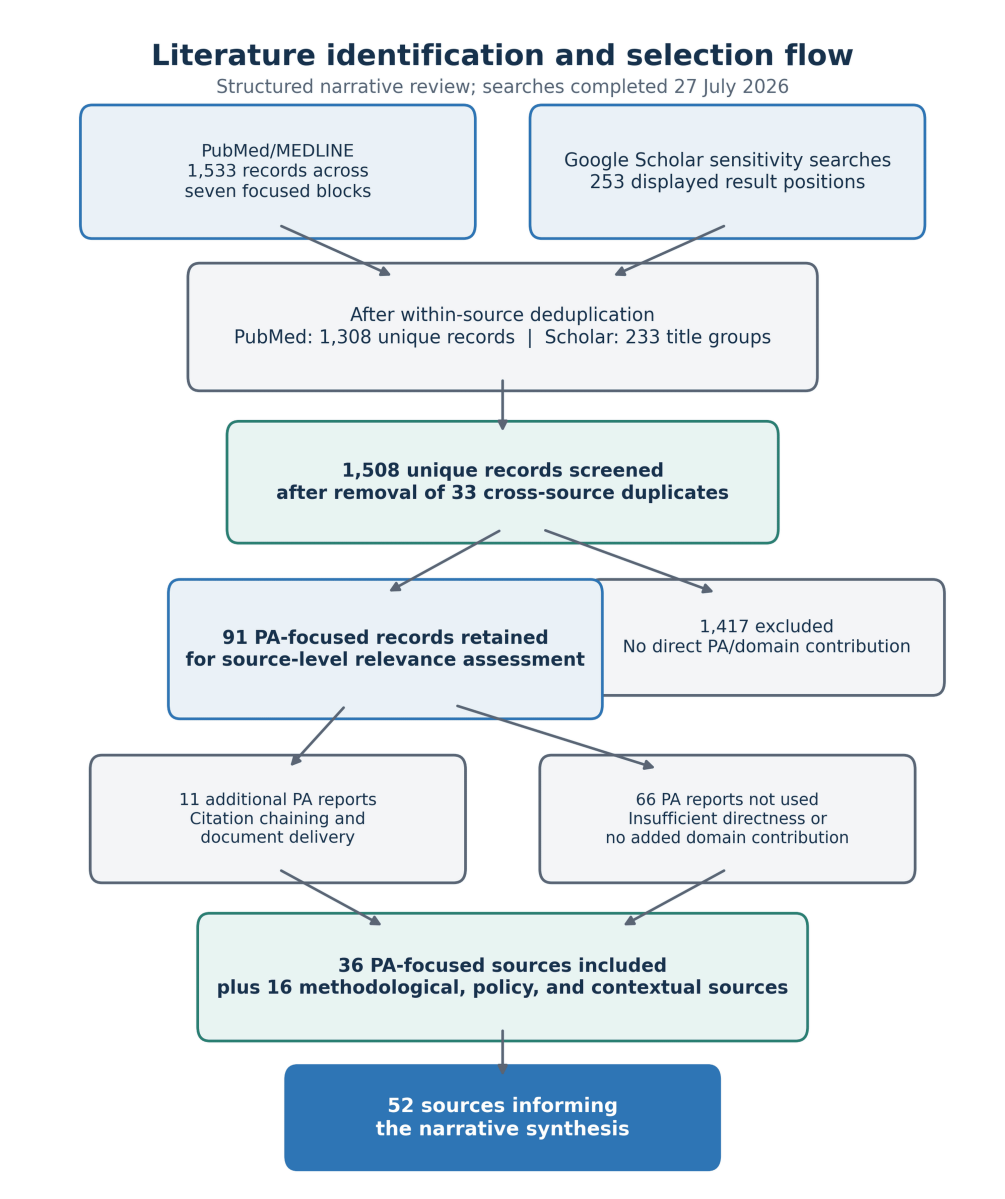
PRISMA-informed literature identification and selection flow for the structured narrative review PRISMA: Preferred Reporting Items for Systematic Reviews and Meta-Analyses; PA: physician assistant Scholar counts represent captured result positions.

The seven PubMed blocks yielded 1,533 records before within-source deduplication and 1,308 unique PubMed records after the removal of 225 duplicate occurrences. The 253 Scholar positions formed 233 unique title groups; 33 additional cross-source duplicates were removed. Thus, 1,508 unique records were screened. Records were eligible when they directly informed PA safety, diagnostic or escalation processes, supervision, onboarding, role understanding, implementation, workload, productivity, cost, or German transferability. English- or German-language empirical studies, reviews, measurement papers, and policy or reporting frameworks were eligible. Education-only reports, non-PA evidence without separable PA findings, unrelated uses of the term assistant, clinical topic papers without workforce outcomes, and sources with no additional domain contribution were excluded. Figure 1 shows the Preferred Reporting Items for Systematic Reviews and Meta-Analyses (PRISMA)-informed selection flow.

All unique titles and available abstracts were assessed against these criteria. Potentially relevant sources were then examined for their contribution to one or more framework domains; full texts were verified for the principal direct studies and for sources from which numerical results were newly extracted. Citation chaining, official German documents, established implementation and quality improvement guidance, and document delivery added sources not captured by the PA-specific database blocks. Because this was a structured narrative review for framework development rather than a comparative effectiveness review, no pooled effect estimate was calculated.

A single risk-of-bias instrument was not applied across the heterogeneous mix of observational studies, qualitative studies, service evaluations, reviews, policy documents, and measurement frameworks. Instead, a structured applicability appraisal recorded study design, population and setting, sample and number of PAs, case-mix selection, supervision ascertainment, outcome definition, principal limitations, and directness for German ambulatory care. This approach can identify threats to interpretation but is not equivalent to a validated design-specific risk-of-bias assessment. Table 2 makes these judgments explicit for the principal direct studies.

**Table 2:** Comparative evidence summary and implications for the proposed framework PA: physician assistant; GP: general practitioner The table emphasizes design, directness, and limitations rather than assigning a common numerical quality score to heterogeneous studies. Source: Original table created by the author.

Study/setting	Design/sample	Finding	Principal limitation	Framework implication
Drennan et al. 2015 [21]/UK general practice	Comparative observational; 2,086 same-day consultations	No adjusted difference in 14-day reconsultation or selected processes; PA visits 5.8 minutes longer	Non-random allocation; different case mix; supervision/sign-off time omitted	Adjust case mix and measure total team time
de Lusignan et al. 2016 [22]/UK primary care	Video observation; 62 consultations, four PAs	All observed consultations rated safe; GPs scored higher across consultation domains	Very small PA sample; selected encounters; limited reliability in some domains	Use direct observation selectively; avoid equivalence claims
Halter et al. 2018 [23]/UK primary care	Case-mix method; 932 PA and 1,154 GP consultations	Patient-level complexity reclassified 30.6% of cases and changed adjusted comparisons	Needs external validation; relies partly on coded records and clinical judgment	Case complexity is a mandatory stratifier
Taylor et al. 2019 [37]/English acute hospitals	Qualitative; 15 patients/patient representatives	Positive communication could coexist with misidentifying PAs as doctors	Small convenience sample across five hospitals	Measure role understanding separately from satisfaction
Joyce et al. 2019 [31]/Irish hospital pilot	Mixed methods; four PAs, staff surveys/interviews	Continuity benefits emerged after integration; organization-wide role awareness remained weak	Single pilot, hospital setting, imported PAs	Track stakeholder communication and role clarity
Edison et al. 2021 [41]/emergency surgery ambulatory care	Service evaluation; 175 referred patients, three months	84% same-day discharge; four grade II or higher complications recorded	No comparator; selected referrals; specialist-supported pathway	Report selection, escalation, follow-up, and complications
Shah et al. 2021 [42]/community mental health	Service evaluation; 72 patients, three months	Structured PA clinic identified cardiometabolic abnormalities and prompted interventions	Incomplete data; uncontrolled; short and incomplete follow-up	Use condition-specific protocols and result-closure measures
Theodoraki et al. 2021 [33]/UK secondary care	Multisource survey; 119 professionals across 13 departments	Most feedback positive; concerns centered on role clarity, workload, and trainee opportunities	Regional voluntary survey; perceptions rather than patient outcomes	Include team effects and training consequences
Moschella and Burrows 2023 [38]/Ontario primary care	Patient survey; 66 respondents in four practices	High trust and access; respondents understood collaboration but preferred physicians for complex problems	Small established-user sample; selection and response bias	Measure access, trust, role understanding, and complexity preference
Burrows et al. 2023 [27]/Ontario mixed settings	Supervisor survey; 93 completed questionnaires	92.9% satisfied; PAs contributed to teaching, quality improvement, and mentorship	Alumni-mediated recruitment; supervisor-reported competence	Measure supervisor experience, team roles, and employment barriers
Showstark et al. 2023/2025 [32,34]/international	Scope-document mapping (25 countries) and scoping review	Marked regional and national heterogeneity in recorded activities and scope	Document availability and terminology vary; activity does not prove outcomes	Do not transfer results without scope and supervision descriptors

Framework development proceeded in four steps. First, variables that repeatedly altered interpretation of PA outcomes were extracted, including diagnostic stage, case complexity, supervision intensity, physician time, escalation, role understanding, and downstream utilization. Second, these variables were mapped to clinical, service, and implementation outcomes [11-13], diagnostic safety concepts [14,15], the Standards for Quality Improvement Reporting Excellence (SQUIRE), and the Standards for Reporting Implementation Studies (StaRI) [16-18]. Third, overlapping variables were consolidated into nine domains. Indicators were assigned to Tier 1 when their absence prevented the interpretation of safety or implementation fidelity; service, economic, and mature-program measures were assigned to Tier 2. Fourth, the proposed minimum dataset and phase-specific pilot were checked for internal consistency across general practice and specialist ambulatory pathways. Formal core outcome set standards require stakeholder consensus and explicit standard-setting procedures; those steps were not undertaken [19].

Comparative evidence and unresolved tensions

The strongest comparative primary-care evidence concerns selected same-day workflows. In 2,086 English consultations, adjusted 14-day reconsultation, tests, referrals, prescriptions, and satisfaction did not differ significantly between PAs and general practitioners, but PA consultations were longer and supervision or prescription-signing time was not included in the apparent cost advantage [20,21]. A subsequent case-mix classification analysis showed that acuity and complexity materially changed classification in 30.6% of cases and affected adjusted comparisons [23]. In a video substudy involving four PAs, all 62 consultations were judged safe, yet general practitioners scored higher across assessed consultation domains [22]. Together, these studies support feasibility under selection and adjustment, not universal equivalence.

Protocolized pathways provide a different signal. A combined PA and senior specialist ambulatory emergency surgery clinic managed 175 referred patients over three months, with same-day discharge for 84% and four Clavien-Dindo grade II or higher complications on follow-up [41]. A PA-led physical health clinic for 72 patients with severe mental illness identified cardiometabolic abnormalities and prompted treatment or follow-up [42]. Both studies show how bounded pathways can generate clinically useful care, but neither was a controlled equivalence study; missing data, referral selection, senior support, and short follow-up limit generalization.

Patient experience is generally favorable but does not resolve role transparency. English hospital patients could report positive communication while mistaking PAs for doctors [37]. In four Ontario family practices, 66 respondents reported trust, access, and understanding of collaboration, but the sample comprised established patients who had already experienced PA care [38]. A co-designed leaflet was feasible and acceptable for introducing PAs to hospital patients, but it has not yet been shown to improve clinical outcomes [39].

Implementation evidence also contains tension. Irish pilot staff valued continuity after working with four PAs, but organization-wide awareness remained poor despite prior communication [31]. Among 119 UK healthcare professionals, most ratings were positive, while negative responses clustered among junior doctors and frequently concerned role clarity, workload, and training opportunities [33]. Ontario supervising physicians reported high satisfaction and broad PA contributions, but salary, billing, and PA supply were barriers [27]. International scope documents and literature show substantial variation in activities and supervision [32,34], limiting the direct transfer of outcome estimates to Germany.

Why conventional PA evaluation is insufficient

The central problem is not simply a shortage of studies but the absence of a common measurement architecture. Reconsultation, consultation duration, satisfaction, documentation quality, tests, referrals, prescriptions, waiting time, throughput, and direct labor cost are all relevant. None is a stand-alone indicator of safe integration, and each can move in a favorable direction while diagnostic risk, physician workload, or downstream use worsens.

A useful endpoint therefore requires a defined exposure, denominator, time window, and clinical context. The same apparent outcome has different meanings in selected post-diagnostic follow-up, supervised same-day primary care, and undifferentiated first-contact care. The evidence in Table 2 supports bounded implementation and measurement of conditional effects; it does not support treating professional title as a sufficient adjustment variable.

Reconsultation is especially vulnerable to misinterpretation. A return may reflect appropriate safety-netting, evolving symptoms, fragmented access, or an incomplete initial assessment; a missed diagnosis may generate no return within the same organization. Reconsultation should therefore be linked to reason, urgency, destination, physician correction, and preventable harm rather than reported as a binary rate.

Patient satisfaction has similar limitations. Positive communication, trust, and access can coexist with incorrect role identification [35-37]. Conversely, a patient may understand the role and still prefer a physician for greater complexity [38]. Satisfaction should be reported alongside role understanding, informed choice, access to physician review, and clinical outcomes.

Safety claims are weakest when no event is reported without encounter counts, active surveillance, follow-up, case risk, or supervision exposure. Reviews have found no UK study designed specifically to detect PA safety incidents and have emphasized the stronger evidence in directly supervised or post-diagnostic settings [9,10]. Table 3 lists the complementary variables required before common endpoints can support claims about safety, efficiency, or value.

**Table 3:** Common PA evaluation outcomes and why they are insufficient when reported alone PA: physician assistant Each endpoint remains useful but requires contextual and complementary measures before supporting claims of safety, efficiency, or value. Source: Original table created by the author.

Common endpoint	What it can show	What it cannot show alone	Required complementary information
Consultation volume	Activity and gross capacity	Appropriateness, diagnostic quality, net physician time released, or downstream work	Case mix, diagnostic stage, supervision time, repeat use, and safety outcomes
Reconsultation rate	Repeated contact within a defined window	Whether return was planned, appropriate, preventable, outside the organization, or related to missed diagnosis	Reason, urgency, destination, adjudicated relationship to index encounter, and 7-, 14-, or 30-day window [20,21]
Patient satisfaction	Reported experience, communication, and acceptability	Role understanding, informed choice, diagnostic accuracy, or safety	Correct role identification, understanding of supervision, request for physician review, and complaints [28,35,36]
No reported adverse events	Absence of detected events under the surveillance method used	True absence of harm when follow-up, denominator, active surveillance, and case risk are unknown	Encounter denominator, follow-up period, incident detection method, case mix, and independent review [9,10,43]
Tests, referrals, or prescriptions	Process and utilization patterns	Appropriateness, clinical necessity, missed disease, or duplication caused by supervision limits	Indication, case-mix adjustment, physician modification, result follow-up, and diagnostic outcome [20,21,40]
Consultation duration	Clinician time per recorded encounter	Total team time, interruptions, sign-off work, or patient time across repeated contacts	PA time, physician time, other staff time, and number of contacts
Direct cost per encounter	Role-specific salary cost multiplied by encounter time	Net cost-effectiveness or cost shifted to physicians and downstream services	Supervision, sign-off, tests, referrals, reconsultations, urgent care, and implementation cost [20,21,44]
Productivity metrics	Work output such as visits, sessions, or relative value units	Safety, role transparency, diagnostic completion, or governance fidelity	Clinical context, complexity, supervision, safety, and patient outcomes [45,46]

Context conditions required for interpretable outcomes

Five contextual descriptors should accompany every PA outcome report: care setting, diagnostic stage, case complexity, supervision model, and PA experience or implementation phase. A reproducible case-mix classification changed patient-level acuity or complexity assignment in 30.6% of 2,086 same-day consultations and altered adjusted comparisons [23]. Professional title alone is therefore an inadequate proxy for clinical exposure. General practice, specialist follow-up, procedural aftercare, and urgent care should not be combined without these descriptors.

Patient safety, diagnostic quality, and escalation

The most favorable evidence is concentrated in bounded workflows. An Irish virtual surgical follow-up service used senior selection, protocols, histopathology review, return triggers, and immediate medical advice for 191 low-risk patients [43]. A specialist-supported ambulatory emergency surgery clinic managed 175 referred patients, with 84% discharged the same day [41]. A PA-led physical health clinic for 72 patients with severe mental illness used a guidance and data collection tool to identify cardiometabolic abnormalities and trigger follow-up [42]. These examples demonstrate feasible pathway measurement, but their selection, supervision, uncontrolled designs, and short follow-up preclude generalization to undifferentiated first-contact care.

Undifferentiated ambulatory presentations require measures capable of detecting diagnostic risk. General practitioners have questioned whether newly qualified PAs recognize limits under complexity and uncertainty [24]. Research on PA contributions to cancer diagnosis has reported tests and referrals but not timeliness of diagnosis [40]. Process completion is therefore not equivalent to diagnostic outcome.

Diagnostic error is a high-priority ambulatory safety problem independent of professional group. One analysis estimated that approximately 5% of US adults experience a diagnostic error in outpatient care each year [14]. A later national burden analysis estimated 795,000 serious misdiagnosis-related harms annually across US care settings, with vascular events, infections, and cancers accounting for a large proportion [15]. These estimates should not be transferred numerically to German PA practice, but they establish why a PA evaluation that omits delayed diagnosis, missed red flags, physician correction, emergency escalation, and unplanned hospital use cannot substantiate diagnostic safety.

Supervision as a safety exposure and measurable resource

Supervision is frequently treated as a fixed background condition although it is both a safety exposure and a resource outcome. Direct observation, physician reassessment, contemporaneous discussion, on-demand availability, remote synchronous support, and retrospective audit are different interventions [25]. Ontario supervisors rated PA competencies and team integration highly, but the survey measured supervisor perceptions and also identified salary, billing, and workforce barriers [27]. UK multisource feedback was predominantly positive while exposing role clarity, workload, and trainee opportunity concerns [33]. Supervision mode and team effects must therefore be measured, not inferred from satisfaction.

The physician work generated by PA integration should be measured rather than assumed. Relevant components include scheduled debriefs, unscheduled interruptions, physician reassessment, review of abnormal results, prescription or order sign-off, documentation review, escalation management, and governance meetings. A lower PA salary or lower direct cost per encounter does not demonstrate cost-effectiveness if these activities are omitted. The same principle applies clinically: a high escalation rate may represent appropriate safety behavior during onboarding, whereas a low escalation rate may reflect either competence or failure to recognize uncertainty. Interpretation requires case mix, phase, and outcome of escalation.

Role transparency and patient experience

Role transparency and patient experience should be separate domains. English patients could value PA communication while believing the PA was a doctor [35,37]. Ontario primary-care respondents described trust, faster access, and understanding of collaboration, yet tended to prefer physicians for more complex problems [38]. A co-designed patient leaflet was feasible and acceptable for introducing the hospital PA role [39]. The minimum dataset should therefore assess correct role identification, understanding of physician responsibility, access to physician review, and satisfaction in that order.

Error communication is also part of transparency and governance. PA-specific guidance recommends joint planning with the supervising physician, transparent disclosure, role-specific education, and avoidance of delegating sole responsibility for disclosure to the PA [47]. Local procedures should specify who investigates, documents, communicates, and follows up adverse events or near misses.

Productivity, cost, and hidden work

Productivity data are useful but highly context dependent. A large Veterans Health Administration analysis found little overall difference between PA and nurse practitioner productivity after adjustment, while specialty, institutional complexity, and geography explained substantial variation [45]. A separate dashboard study proposed operational indicators such as work relative value units, visits, encounter type, collections, and session utilization [46]. These measures can show activity and resource use, but not whether the activity was appropriately selected, safely supervised, diagnostically complete, or understood by patients.

The same caution applies to economic reviews. International evidence suggests potential cost advantages in some contexts, but results depend on salary structures, task substitution, case mix, physician input, and downstream utilization [44]. German ambulatory care adds billing and delegation constraints that differ from US and UK systems. Economic reporting should therefore use a net-resource perspective: PA time plus physician supervision time, additional staff coordination, repeated contacts, tests, referrals, and downstream urgent care. Cost per PA encounter alone is an accounting measure, not a value outcome.

Implementation outcomes and continuous quality improvement

PA integration is a complex service intervention. Acceptability and adoption do not establish fidelity, safety, or sustainability [11,13]. In Ireland, staff who worked with four newly introduced PAs valued continuity, but hospital-wide awareness remained poor despite prior communication [31]. International scope documents and literature show marked variation in recorded responsibilities and supervision [32,34]. Implementation measures must therefore capture patient selection, scope adherence, supervision availability, role communication, escalation criteria, and effects on other team members.

A practical evaluation should therefore combine three layers. The first layer asks whether implementation occurred as intended: appropriate patient selection, scope adherence, supervision availability, and use of escalation criteria. The second assesses service consequences: access, waiting time, continuity, workflow, physician workload, and cost. The third assesses patient and clinical outcomes: harm, diagnostic correction, delayed diagnosis, emergency use, patient understanding, and experience. Continuous quality improvement methods can then use repeated data review, feedback, and Plan-Do-Study-Act cycles to refine triage, scheduling, escalation, and supervision [16-18].

German transferability and governance

German evidence remains limited and mostly descriptive. A national survey of 169 PAs found broad variation in activities and weaker ratings for development opportunities [5]. A small, largely hospital-based study reported satisfaction and broad activity profiles but was not designed to establish ambulatory safety or cost-effectiveness [6]. A recent German outpatient review described potential benefits alongside unresolved legal, organizational, refinancing, and professional boundary issues [7]. These sources strengthen the case for context-specific evaluation but do not supply German effect estimates.

These findings argue against a single productivity target or binary judgment that PA integration is safe or unsafe. Germany should evaluate defined clinical pathways. A stable post-procedural review and undifferentiated same-day symptoms require different case selection and supervision assumptions. The common requirements are measurable selection, supervision, escalation, role transparency, downstream follow-up, and accountability.

Proposed core outcome framework

The proposed framework in Table 4 comprises nine domains. It is an author proposal derived from the evidence-to-domain mapping described above, not a finding that the literature has validated these domains. Tier 1 contains the minimum safety and implementation variables required to interpret an evaluation. Tier 2 adds service, economic, workforce, and patient outcomes for formal research or mature programs. A site should not describe an evaluation as safety-oriented when Tier 1 denominators and surveillance are absent.

**Table 4:** Proposed core outcome framework for PA integration in German ambulatory care PA: physician assistant Tier 1 indicators are the proposed mandatory minimum for a safety-oriented evaluation. Tier 2 indicators support mature programs or formal research. Source: Original table created by the author.

Outcome domain	Core definition	Tier 1 mandatory indicators	Tier 2 extended indicators and interpretation
Patient and diagnostic safety	Harm or potential harm associated with delegated care or failure of the care pathway	Adverse events; near misses; delayed or missed diagnosis detected through active surveillance; unplanned emergency referral or hospital admission; denominator and follow-up interval	Preventability and severity review; trigger tool chart review; complaint analysis. Zero events are interpretable only with active surveillance [9,10,14,15]
Diagnostic process quality	Completeness and appropriateness of information gathering, assessment, plan, and result follow-up	Physician modification of PA assessment or plan; direct reassessment; abnormal result closure; documentation completeness	Blinded chart review; diagnostic concordance; time to definitive diagnosis; disease-specific process indicators [22,40]
Escalation and red-flag handling	Recognition of uncertainty or predefined risk and timely transfer to physician decision-making	Escalation rate by trigger; time to physician input; disposition after escalation; failure to escalate identified on review	Sensitivity of local trigger rules; appropriateness adjudication; trend by experience phase. High early escalation may reflect safe onboarding [24,25]
Supervision burden and hidden physician work	Physician resources required to support, correct, authorize, and govern delegated care	Scheduled debrief minutes; unscheduled interruptions; direct reassessments; sign-off tasks; named supervisor availability	Governance and training time; supervision cost; distribution across physicians; perceived burden and psychological safety [8,24-26,29,30]
Role transparency and patient understanding	Patient knowledge of the professional role, limits, and access to physician review	Correct identification of PA versus physician; understanding that care is delegated; knowledge of how to request physician review	Patient choice, information recall, complaints about role confusion, and subgroup differences [28,35,36]
Patient experience and satisfaction	Patient-reported experience of access, communication, involvement, confidence, and continuity	Brief validated or locally consistent experience items; willingness to see the PA again; complaints	Qualitative interviews; continuity preference; trust and perceived competence. Report separately from role understanding [35,36,48]
Workflow, access, continuity, and productivity	Effects on service capacity and movement of work across the team and care pathway	Appointments delivered; waiting time; consultation duration; reconsultations; continuity; physician time released net of supervision	Session utilization, task completion, pathway delays, staff redistribution, and specialty-adjusted productivity [8,20,21,45,46]
Economic and resource impact	Net resource consequence across the practice and downstream services	PA employment cost; physician supervision cost; other staff time; tests; referrals; repeat contacts	Incremental cost-effectiveness; patient time and travel; downstream urgent and hospital care; sensitivity analysis [20,21,43,44]
Implementation fidelity, governance, team effects, and sustainability	Whether the role was implemented as intended, remained within scope, and became supportable over time	Written delegation matrix; competence records; pathway adherence; supervision delivered as planned; event review process; staff role clarity	Acceptability, adoption, appropriateness, feasibility, penetration, sustainability, retention, effects on physician training, and interprofessional collaboration [5,6,11,13,16,47,49-52]

**Figure 2:**
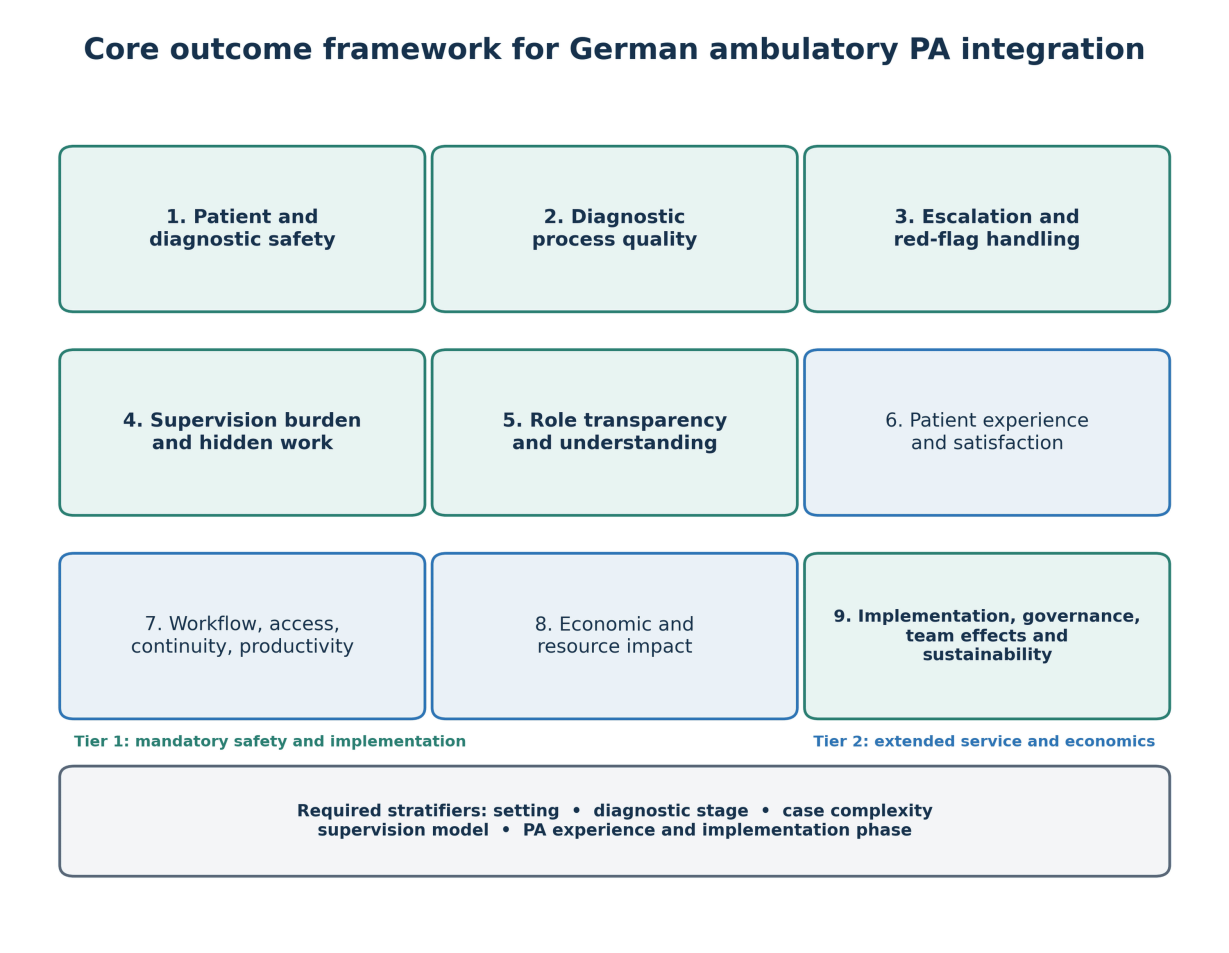
Architecture of the proposed framework PA: physician assistant Green domains constitute the proposed Tier 1 safety and implementation core; blue domains are Tier 2 extensions. All outcomes require the five contextual stratifiers.

The framework deliberately avoids a composite score because aggregation could conceal a rare serious harm behind modest access or satisfaction gains. Results should instead be displayed by domain and interpreted with the five contextual stratifiers in Figure 2.

Operational supervision terminology

Operational terminology is necessary because the word supervision is used inconsistently. Table 5 defines six modes that can be documented at encounter or pathway level. Direct observation and direct physician assessment provide the greatest contemporaneous oversight but consume more physician time. Contemporaneous case review allows the physician to modify the plan before final disposition without necessarily repeating the entire encounter. On-demand on-site supervision may be appropriate for an experienced PA in a low-risk protocolized pathway. Remote synchronous support is real-time but is not equivalent to physical reassessment. Retrospective audit is a quality improvement activity and should never be reported as if it protected the patient during the index encounter.

**Table 5:** Operational terminology for supervision in German ambulatory PA care PA: physician assistant Retrospective audit supports quality improvement but is not contemporaneous clinical supervision. All modes remain within physician-led delegation in Germany [2-4]. Source: Original table created by the author.

Supervision mode	Operational definition	Typical use	Minimum documentation
Direct observation	The physician observes the PA-patient interaction or relevant examination or procedure component in real time	Onboarding, new competencies, procedural training, or selected high-risk learning encounters	Observer, task, findings, feedback, and competence decision
Direct physician assessment	The physician independently assesses the patient before final diagnosis, treatment, referral, or discharge	Red flags, diagnostic uncertainty, deterioration, abnormal vital signs, complex multimorbidity, or physician-reserved decisions	Physician examination, final assessment, plan, and responsibility
Contemporaneous case review	The PA and physician discuss or review the case before final disposition; the physician may modify the plan without repeating the full encounter	Moderate-risk or non-standard findings, abnormal results, or uncertainty in an otherwise delegable pathway	Time, physician, issue discussed, and agreed or modified plan
On-demand on-site supervision	A named physician is physically available and reviews the patient when a trigger is met or the PA requests input	Experienced PA, stable low-risk follow-up, and protocolized care with explicit escalation criteria	Named supervisor, planned model, triggers assessed, and any escalation
Remote synchronous support	A named physician provides real-time audio or audiovisual input but is not physically present	Defined remote or telemedical pathways when local policy and clinical risk permit	Modality, availability, discussion, limitations, and contingency for physical assessment
Retrospective audit	Records or outcomes are reviewed after the encounter for learning, fidelity, or quality assurance	Periodic sample review, event analysis, documentation audit, and pathway improvement	Sample, reviewer, findings, corrective action, and review date. Not recorded as supervision of the index encounter [25]

For German ambulatory care, all six modes remain nested within physician-led delegation. The appropriate mode should be determined by the task, diagnostic stage, risk, PA experience, and local resources. Studies should report the planned supervision model, the model actually delivered, and reasons for deviations.

Minimum dataset for German ambulatory PA pilots

The minimum dataset in Table 6 is designed for routine feasibility rather than exhaustive research. It begins with encounter denominators and the five contextual descriptors. It then captures safety signals, diagnostic and escalation processes, physician supervision time, role understanding, patient experience, access and continuity, resource use, and governance fidelity. Each variable has a defined source and measurement interval.

**Table 6:** Minimum dataset and measurement schedule for German ambulatory PA pilots PA: physician assistant The dataset should be linked at the encounter level where possible and summarized as a domain dashboard rather than a single composite score. Source: Original table created by the author.

Variable family	Minimum variables	Primary source	Timing	Interpretation rule
Context and denominator	Encounter and patient counts; setting; pathway; diagnostic stage; risk/complexity; PA experience phase; planned supervision	Scheduling system and structured encounter field	Every encounter; monthly summary	All rates require a stated denominator and contextual stratification
Safety surveillance	Adverse event, near miss, delayed/missed diagnosis, complaint, unplanned emergency referral, admission, death; severity and relation to PA pathway	Incident system, trigger chart review, complaints, linked records	Continuous; monthly review; immediate serious event review	Zero events require an active detection method and follow-up interval
Diagnostic process	Physician modification; direct reassessment; abnormal result closure; documented differential/uncertainty; revised diagnosis	Clinical record and review sample	Every triggered case; monthly sample	Correction is not automatically failure; assess timing, preventability, and patient effect
Escalation	Trigger present; escalation initiated; time to physician input; final disposition; missed trigger on audit	Structured PA note and physician confirmation	Every encounter with trigger; monthly aggregate	Interpret by case mix and experience phase
Supervision and hidden work	Scheduled debrief, unscheduled interruption, reassessment, sign-off, record review, governance time	Time log or electronic quick entry field	Daily or weekly log; monthly aggregate	Calculate net physician time released, not gross PA activity
Role transparency	Patient correctly identifies role; understands delegation and physician availability; requested physician review	Two- or three-item post-visit survey and complaints	Sample monthly and after communication changes	Report separately from satisfaction
Patient experience	Access, communication, involvement, confidence, continuity, willingness to return	Short patient survey	Sample monthly or quarterly	Avoid interpreting satisfaction as evidence of safety
Workflow and continuity	Waiting time, consultation duration, completed pathway contacts, 7-/14-/30-day reconsultation, continuity, referral and test patterns	Scheduling and clinical records	Monthly; baseline comparison	Classify planned versus unplanned and same versus linked problem
Economic and resource use	PA cost, physician supervision cost, other staff time, tests, referrals, repeat contacts, urgent/hospital use	Payroll, time logs, records, claims where available	Quarterly after stabilization	Do not claim cost-effectiveness from direct encounter cost alone
Implementation and governance	Delegation matrix, competence assessment, protocol fidelity, planned versus delivered supervision, staff role clarity, retention, event review completion	Governance audit and staff survey	Baseline, 12 weeks, six months, and then quarterly/annually	Scope expansion requires evidence of competence, fidelity, and stable safety indicators

At minimum, every PA encounter dataset should identify the setting, pathway, diagnostic stage, case risk category, PA experience phase, and planned supervision level. Outcomes should use an encounter denominator and, where relevant, a patient denominator to avoid distortion from repeated attenders. Safety events should be actively sought through incident logs, trigger-based chart review, complaints, unplanned emergency or hospital use, and structured review of selected reconsultations. A finding of zero events should state the surveillance method and follow-up interval.

Diagnostic quality should be assessed with measures that are feasible and interpretable. These may include physician modification of the assessment or plan before disposition, direct physician reassessment, abnormal result follow-up, escalation for predefined red flags, unplanned reconsultation within seven, 14, or 30 days, emergency referral, hospital admission, and delayed or revised diagnosis. Reconsultations should be adjudicated in samples rather than treated automatically as failure. The objective is not to penalize escalation but to distinguish appropriate safety behavior from missed risk or duplicated work.

Supervision should be time-stamped whenever feasible. A simple electronic or weekly log can record scheduled debrief time, unscheduled interruptions, physician reassessment, sign-off tasks, and governance time. This enables the calculation of net physician time released rather than gross PA encounter volume. Patient role understanding can be measured with a two- or three-item post-visit survey asking who provided the consultation, whether that person was a physician, and whether physician review was available. These questions should precede satisfaction questions to reduce cueing.

Economic evaluation should be delayed until the pathway is stable enough for meaningful comparison. Early onboarding is expected to consume supervision and training resources. Mature evaluations should include PA employment cost, physician time, other staff coordination, tests, referrals, repeated visits, urgent care, and hospital use. Productivity should be reported alongside safety and supervision, not as a substitute for them.

Phase-specific pilot evaluation model

A prospective pilot can be implemented in four phases. During a four- to eight-week baseline phase, the practice should measure existing access, physician workload, case mix, reconsultations, referrals, tests, safety events, and patient experience for the pathway that will later involve a PA. During the first 12 weeks of onboarding, the site should use a written scope, named supervisors, reduced or graduated scheduling, frequent debriefs, observed competence assessments, and explicit escalation triggers. Onboarding research supports competence-focused teaching, electronic health record training, mentorship, organizational orientation, role clarification, and a tailored increase in patient volume [29]. Primary-care preceptorship and supervision reports likewise describe high initial supervision that can reduce as competence and local familiarity develop [26,30].

During stabilization, usually months 3-6, the site should compare pathway performance with baseline and assess whether supervision can be adjusted without deterioration in safety or diagnostic process indicators. During sustainment, usually months 6-12 and thereafter, the focus should expand to penetration, staff retention, economic consequences, and whether governance remains functional. A mature pathway should still undergo quarterly review and event-triggered reassessment of scope.

Randomization is not essential for initial local quality evaluation, but design limitations must be explicit. Options include prospective before-and-after evaluation, controlled comparison with a similar pathway, interrupted time series, or stepped implementation across sites. Analyses should adjust or stratify for age, multimorbidity, presenting problem, urgency, diagnostic stage, and socioeconomic factors when sample size permits. Small pilots should emphasize transparent descriptive data and run charts rather than underpowered claims of non-inferiority.

Predefined interpretation rules are more useful than universal numerical thresholds. For example, a rise in escalation during early onboarding may indicate appropriate detection of uncertainty; the same rise after pathway stabilization may signal case selection drift, training needs, or worsening population complexity. A decline in physician interruptions may indicate growing competence, but only if direct reassessment, delayed diagnosis, and unplanned use do not worsen. Monthly multidisciplinary review should examine linked patterns rather than isolated metrics and use quality improvement cycles to modify triage, protocols, documentation, or supervision [16-18].

Interpretation and implementation implications

The principal implication is that measurable safety is not synonymous with an absence of reported incidents. A safe implementation is one that selects appropriate patients, detects uncertainty, escalates reliably, provides timely physician input, documents accountability, informs patients about professional roles, and learns from deviations. Some of these processes consume time. Their presence should not automatically be classified as inefficiency; they are part of the safety mechanism whose cost and benefit must be evaluated.

The framework also changes how claims should be phrased. An increase in appointments demonstrates increased activity. High satisfaction demonstrates a positive reported experience. A lower direct labor cost demonstrates an accounting difference. None alone demonstrates safe substitution, net physician relief, diagnostic equivalence, or cost-effectiveness. Stronger conclusions require convergence across safety, diagnostic, supervision, patient, workflow, economic, and implementation domains.

For policy and multicenter research, standardization of the five contextual descriptors is at least as important as standardization of endpoints. A study of a protocolized wound check and a study of undifferentiated chest pain should not be pooled merely because both encounters were conducted by PAs. Reporting diagnostic stage, complexity, supervision, and experience would make future evidence more comparable and reduce inappropriate transfer between jurisdictions or settings.

The modular Tier 1/Tier 2 architecture is intended to preserve this cross-setting applicability. Tier 1 provides a non-negotiable safety and governance floor across general and specialist ambulatory care, whereas Tier 2 allows services to add pathway-specific indicators without distorting the common core. Although developed for PA integration, several domains may also inform evaluation of other physician-led delegated care models, provided that profession-specific legal scope and competency requirements are reported separately.

The framework can also support professional development. Onboarding indicators can identify when a PA is ready to move from direct observation to contemporaneous review or on-demand supervision for a defined task. Governance indicators can show whether expanded activity is accompanied by competence evidence, protocol adherence, and stable safety outcomes. In this way, measurement becomes a mechanism for calibrated delegation rather than surveillance for its own sake.

Team effects deserve deliberate measurement. Interprofessional collaboration can improve professional practice and service outcomes, but unclear boundaries, inadequate communication, or competition for training opportunities can undermine implementation [51,52]. Staff surveys should therefore assess role clarity, psychological safety for escalation, supervisor burden, and effects on physician training. These outcomes are particularly important in practices where the same physicians supervise PAs, medical trainees, and other delegated staff.

Limitations

This structured narrative review used PubMed/MEDLINE as the only bibliographic database and Google Scholar only as a supplementary sensitivity source. Embase, Scopus, Web of Science, and CINAHL (Cumulative Index to Nursing and Allied Health Literature) were not searched; relevant records may therefore have been missed. Scholar retrieval is affected by opaque ranking and unstable approximate totals. Restriction to English- and German-language full texts creates language bias, and publication bias may favor successful implementations. Screening and synthesis were conducted within a single-author review with organizational assistance from a language model, so independent duplicate screening was not available.

The framework combines direct PA evidence with indirect diagnostic safety, implementation science, and quality improvement sources. No single validated risk-of-bias tool was applied across heterogeneous source types; the structured applicability appraisal in Table 2 does not replace design-specific critical appraisal. Most comparative evidence comes from the United Kingdom, the United States, Canada, or the Netherlands, where education, prescribing, financing, regulation, and supervision differ from Germany. Several studies involved few PAs, selected case mix, supervisor-reported outcomes, or short follow-up.

The proposed framework has not undergone Delphi consensus, patient-priority setting, psychometric validation, or prospective feasibility testing. Stakeholders were not involved in selecting domains or tier assignments. Measures such as preventability, diagnostic delay, and supervision time require local operational definitions and may create documentation burden. The next steps are a German multi-stakeholder consensus process, prospective pilots in general and specialist ambulatory care, feasibility and reliability testing, and refinement of a parsimonious electronically supported Tier 1 dataset.

## Conclusions

The evidence supports the feasibility and acceptability of PAs in selected, supervised, and often protocolized pathways, but it does not establish safety, equivalence, or net physician relief across undifferentiated German ambulatory care. Case mix, diagnostic stage, supervision, escalation, role understanding, and downstream utilization determine how conventional outcomes should be interpreted.

The nine-domain framework and minimum dataset are the author's proposed response to these evidence gaps, not a validated core outcome set. Their immediate purpose is to make German pilot evaluations transparent and comparable. Multi-stakeholder consensus and prospective validation are required before the framework can define benchmarks, support policy thresholds, or justify claims of safe substitution.

## Figures and Tables

**Figure 3:**
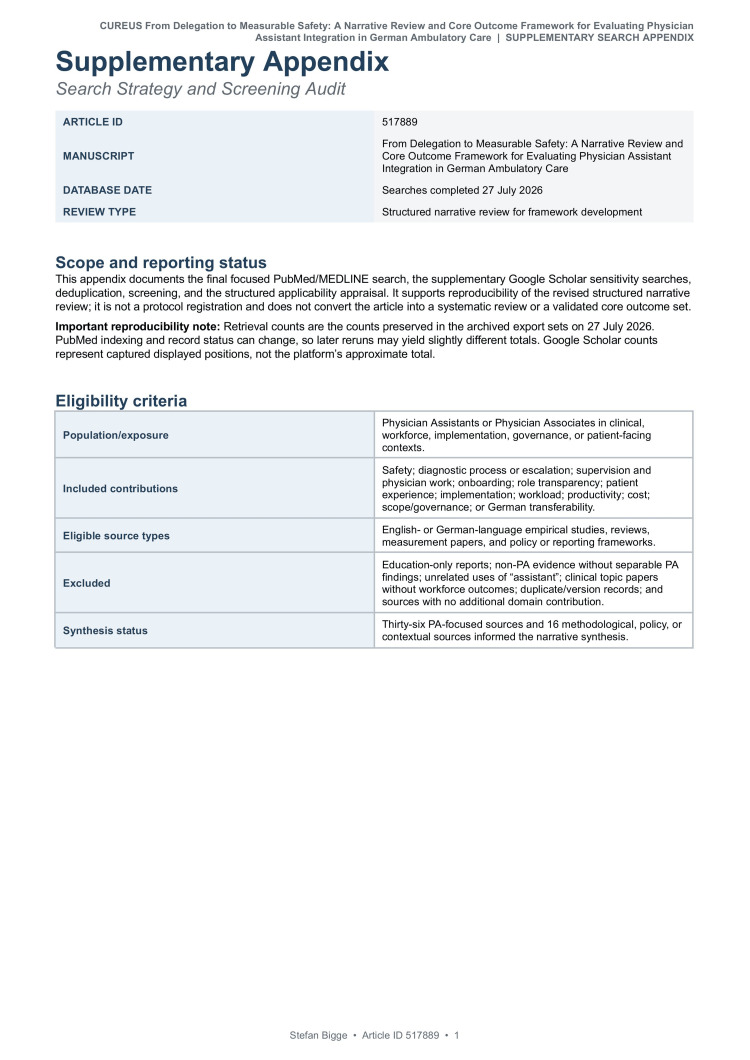
Full reproducible syntax

**Figure 4:**
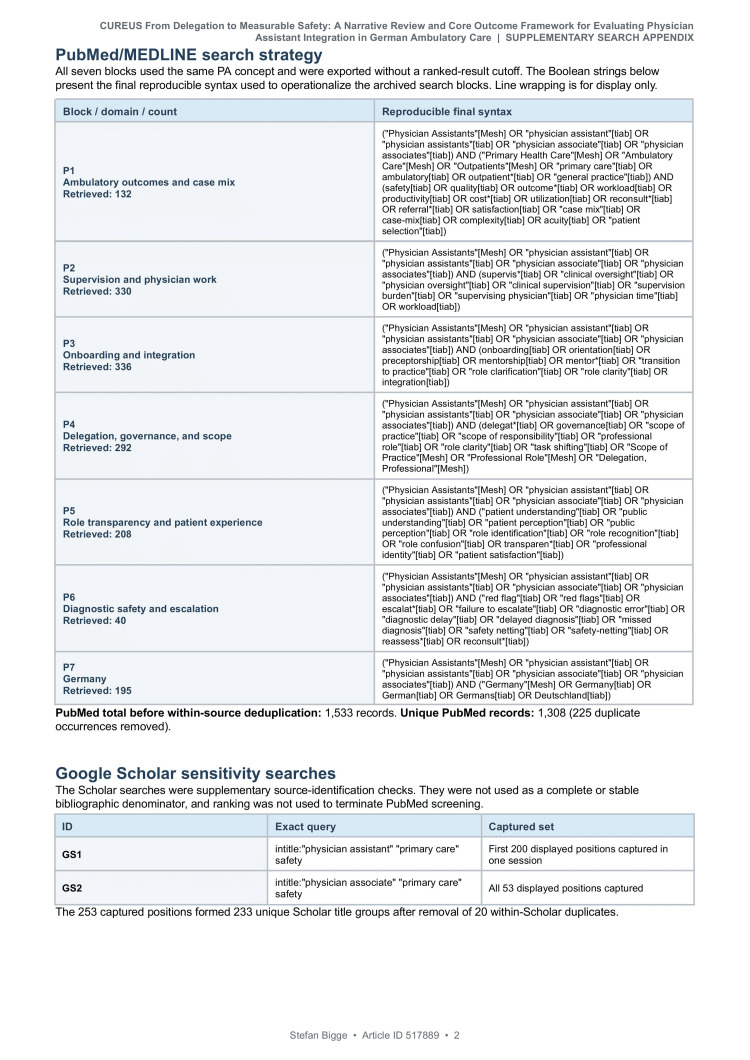
Full reproducible syntax (continuation)

**Figure 5:**
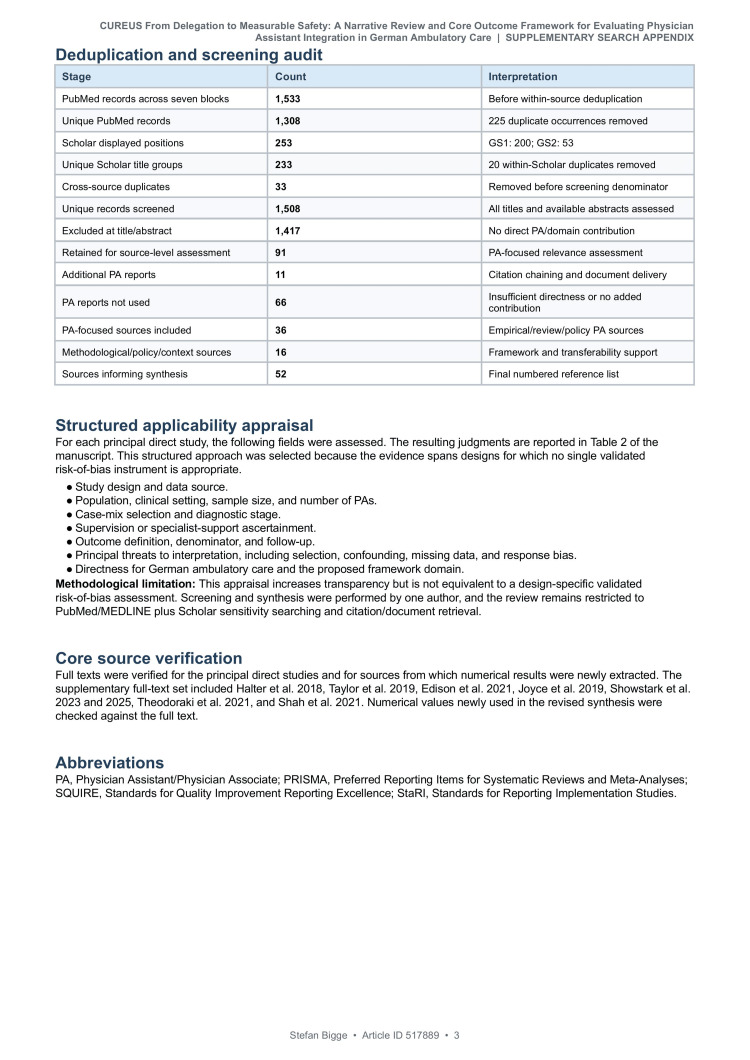
Full reproducible syntax (continuation)
